# Outcomes of combined single-use dual blade goniotomy and cataract surgery

**DOI:** 10.1007/s10792-022-02257-x

**Published:** 2022-03-31

**Authors:** Sabine Baumgarten, Niklas Plange, Hla Myint Htoon, Tibor Lohmann, Andreas Videa, Antonis Koutsonas, Hannah Schellhase, David Kuerten, Peter Walter, Matthias Fuest

**Affiliations:** 1 Department of Ophthalmology, RWTH Aachen University, Pauwelsstrasse 30, 52074 Aachen, Germany; 2 Augenzentrum Am Annapark, Steigerweg 3, 52477 Alsdorf, Germany; 3 Singapore Eye Research Institute, 11 Third Hospital Ave, Singapore City, 168751 Singapore; 4 Duke-NUS Medical School, 8 College Road, Singapore City, 169857 Singapore

**Keywords:** Glaucoma, Intraocular pressure, Single-use dual blade goniotomy, Kahook Dual Blade goniotomy

## Abstract

**Purpose:**

Single-use dual blade goniotomy (SBG) is a novel ab interno procedure that removes three to five clock hours of trabecular meshwork (TM). We analysed the reduction of intraocular pressure (IOP) and topical glaucoma medication (Meds) in eyes following combined cataract surgery and SBG (Cat-SBG).

**Methods:**

IOP and Meds were evaluated retrospectively in 55 eyes of 38 patients. 44 eyes had high tension glaucoma (HTG) and eleven eyes had normal tension glaucoma (NTG). Complete success (no Meds) and qualified success (with Meds) for IOP levels ≤ 21, ≤ 18 , ≤ 16 mmHg or ≥ 20% IOP reduction at the two- and six-month follow-up were evaluated.

**Results:**

IOP and Meds were significantly reduced from before to two months after Cat-SBG in HTG- and NTG-patients (HTG: IOP 19.4 ± 3.3 to 15.1 ± 3.3 mmHg; *p* < 0.001; Meds 2.1 ± 1.3 to 0.8 ± 1.3; *p* < 0.001; NTG: IOP 14.0 ± 2.3 to 11.5 ± 2.3 mmHg; *p* = 0.004; Meds 1.6 ± 0.7 to 0.3 ± 0.7; *p* < 0.001). IOP and Meds did not change significantly from two to six months after Cat-SBG.

In HTG, complete and qualified success rates were 43% (19/44) and 93% (41/44) for IOP ≤ 18 mmHg, 36% (16/44) and 64% (28/44) for IOP ≤ 16 mmHg and 30% (13/44) and 43% (19/44) for ≥ 20% IOP reduction six months after surgery. In NTG, complete and qualified success was 81% (9/11) and 100% (11/11) for IOP ≤ 18 and ≤ 16 mmHg, and 27% (3/11) for IOP reduction ≥ 20%.

IOP and Meds reduction were comparable between HTG and NTG eyes.

Only minor postoperative complications occurred.

**Conclusion:**

Cat-SBG is an efficient method to significantly lower IOP in patients with HTG and NTG.

## Introduction

Glaucoma is a leading cause of blindness [1], and the reduction of intraocular pressure (IOP) is considered to decelerate its progression [2, 3]. Obstruction of aqueous outflow at the level of juxtacanalicular trabecular meshwork (TM) and distal outflow structures is a major component of IOP dysregulation [4, 5]. Performing trabeculotomy or goniotomy in adults with glaucoma showed variable success rates [6, 7]; an incomplete removal of TM and membrane formation across the remaining TM leaflets leading to subsequent IOP elevation has been reported [6–8].

Single-use dual blade goniotomy (SBG) is a minimally invasive glaucoma surgery (MIGS) that removes an approximately 230 µm wide strip of TM with two blades via an ab interno approach [9, 10]. Three to five clock hours of TM circumference are removed so that the resistance of the aqueous outflow pathway is decreased [11, 12]. Compared to traditional ab externo trabeculotomies, a complete excision of the TM tissue is achieved instead of only an incision [11, 13]. By minimizing residual TM leaflets, the development of fibrosis is reduced and thus, SBG potentially surpasses outcomes of traditional ab externo trabeculotomy in adults [12]. Recent studies showed convincing success rates for the SBG procedure at reducing IOP and glaucoma medication (Meds) as a standalone procedure or combined with cataract surgery [12, 14–16].

While Cat-SBG has shown good results in patients with HTG [15, 17, 18], there are currently no data on the effect in NTG patients.

In this study, we examined Cat-SBG outcomes performed at two specialized centres and analysed success rates in patients with mild to advanced high-tension glaucoma (HTG) as well as in patients with normal tension glaucoma (NTG).

## Patients and methods

Included in this retrospective study were 55 eyes of 38 patients. 44 eyes of 31 patients had HTG (primary open-angle glaucoma (POAG, *n* = 33), pseudoexfoliation glaucoma (PEX, *n* = 11)). 16 HTG patients were female, 15 male and 23 right eyes and 21 left eyes were included. The mean age of HTG patients was 74 ± 9 years (49 to 87 years).

Eleven eyes of seven patients had NTG. Three NTG patients were female and four were male. The mean age of NTG patients was 68 ± 12 years (49 to 81 years).

Patients had mild to advanced glaucoma according to Hodapp et al. [19] (mild = 40, moderate = 10, advanced = 5). Visual field defects were documented by static computerized perimetry, using the Humphrey 24–2 Swedish Interactive Thresholding Algorithm (SITA) program (Carl Zeiss Meditec, Jena, Germany). MD was − 8.1 ± 6.8 dB (− 22.6 to − 0.18 dB) in HTG eyes and − 5.9 ± 11.4 dB (− 23.0 to − 0.25 dB) in NTG eyes.

All eyes underwent combined Cat-SBG. All procedures were performed following a standardized protocol between August 2017 and May 2020. SBG was only performed after uneventful cataract surgery. Indication for Cat-SBG was the aim to reduce the dependence on IOP lowering eye drops and to improve the IOP level. The procedure was recommended in all glaucoma patients taking IOP lowering eye drops or ocular hypertension planned for cataract surgery.

Meds, IOP values and complications were evaluated two and six months after Cat-SBG. Complete success, referring to patients that reached the target IOP without Meds, and qualified success, reaching target IOP irrespective of Meds, were evaluated at the two- and six-month follow-up for IOP ≤ 21, ≤ 18 and ≤ 16 mmHg as well as ≥ 20% IOP reduction.

The Institutional Ethical Review Board of the RWTH Aachen University approved the study (EK 410/20). The described research adhered to the tenets of the Declaration of Helsinki.

## Surgical technique

Phacoemulsification (phaco) with in the bag implantation of a foldable acrylic lens (CT Asphina 409MP, Carl Zeiss Meditec, Jena, Germany) was performed under regional (topical and intraocular) anaesthesia.

**Fig. 1:**
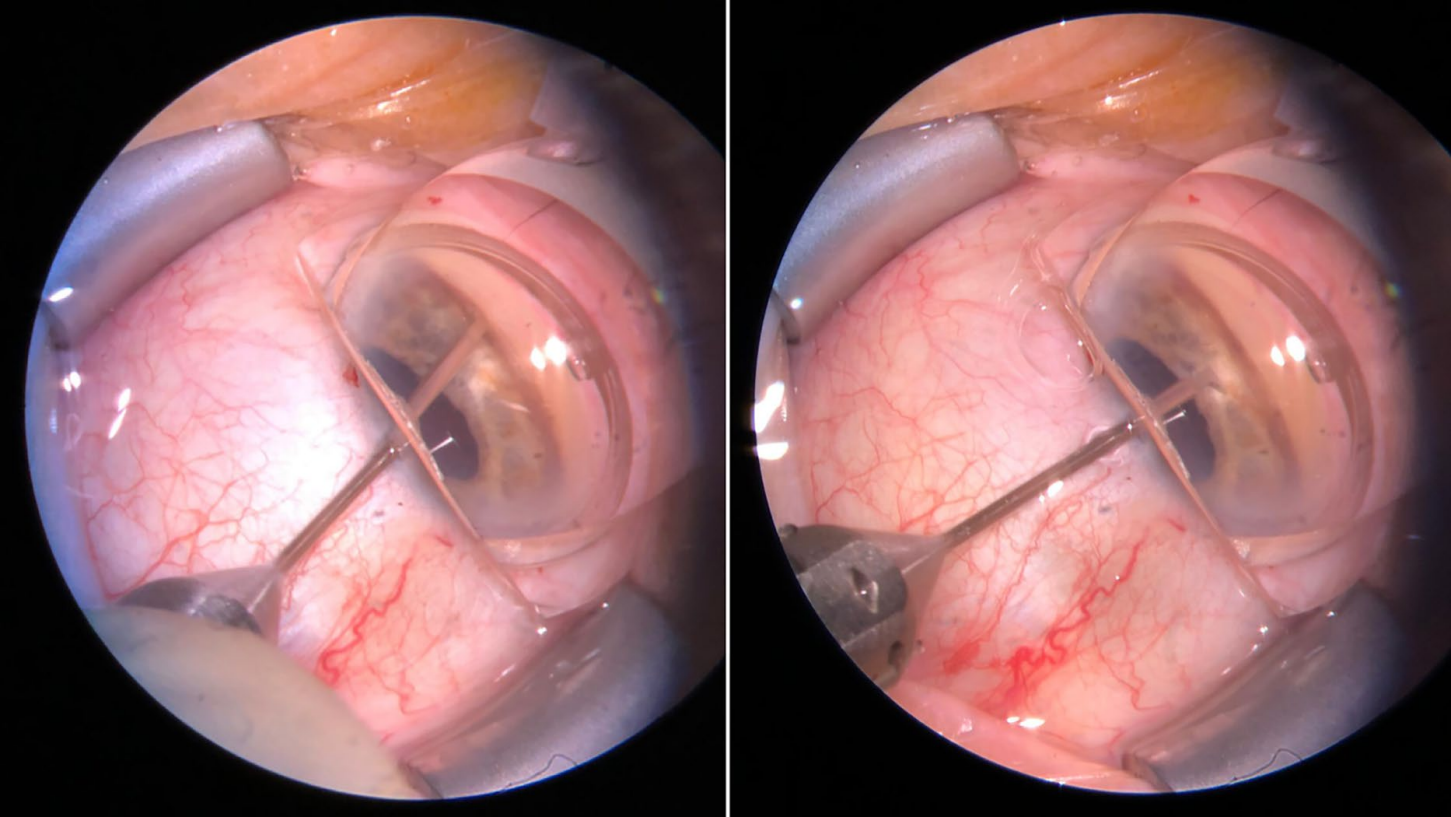
Intraoperative set-up of combined cataract surgery with single-use dual blade goniotomy (Cat-SBG): the single-use dual blade (SDB) is cutting the trabecular meshwork (TM) nasally for three to five clock hours

Then, to constrict the pupil, acetylcholine chloride 1% (Miochol-E, Dr. Gerhard Mann, Berlin, Germany) was instilled into the anterior chamber. To deepen the chamber angle, a cohesive viscoelastic (sodium hyaluronate 1%, Healon Pro, Johnson & Johnson, New Brunswick, USA) was injected into the nasal anterior chamber (AC) angle. The angulation of the microscope (OPMI VISU 210, S8, Carl Zeiss Meditec, Jena, Germany) was adjusted, and the patient’s head tilted to allow visualization of the nasal AC angle through a single-use gonioprism lens (MV LV 48, Phakos, Montreuil, France). The blade (Kahook Dual Blade, New World Medical, Rancho, CA, USA) was introduced through the temporal paracentesis initially used for cataract surgery. The TM excision with the single-use dual blade (SDB) was started at the midnasal 3/9 o’clock position and then advanced inferiorly (Fig. 1). The sharp tip of the SDB was inserted through the TM and into Schlemm’s canal (SC). The heel of the SDB was seated against the wall of SC, and the blade was advanced within the canal. Then, the SDB was slightly withdrawn, turned and another cut performed from the 3/9 o´clock position superiorly. Total TM excision was three to five clock hours. In this way, two strips of TM were generated. In case of a large, mobile strip, a 23G intraocular forceps (Geuder AG, Heidelberg, Germany) was used to remove it through the temporal paracentesis to avoid incarceration (n = 2). After flushing out the viscoelastic from the anterior chamber, acetylcholine chloride 1% was injected again to further constrict the pupil. The corneal wounds were hydrated and checked for water-tight closure. At the end of the operation, IOP was set at a slightly elevated level, aiming for 20-25 mmHg, to prevent blood reflux into the AC. To reduce the risk of postoperative (postop) fibrinous reaction and consecutive posterior synechia, an air bubble (50% of anterior chamber) was instilled at the end of the surgery.

After surgery, patients were treated with topical antibiotics (ofloxacin 0.3%, Floxal EDO, Dr. Gerhard Mann, Berlin, Germany) and prednisolone acetate 1% eye drops (Inflanefran forte, Allergan, Frankfurt am Main, Germany) every two hours for one week, followed by three times daily until for two weeks postoperatively to avoid infection and inflammation.

## Statistical analysis

All values were expressed as the mean ± standard deviation (range: minimum to maximum).

Statistical analysis was performed using IBM SPSS Statistics for Macintosh, Version 26.0, Armonk, NY: CBM Corp. The changes in IOP and Meds over time were investigated using a linear mixed model to account for fellow-eye correlations (utilizing an autoregressive first-order correlation co-variance structure based on smaller the better, Hurvich’s & Tsai’s (AICC) information criterion), and Bonferroni’s correction was used to address multiplicity. Comparisons between categorical variables were conducted using the Fisher’s exact test. According to Kolmogorov–Smirnov tests, all parameters were identified as normally distributed. A *P*-value of < 0.05 was considered statistically significant.

## Results

A summary for patients’ characteristic is presented in Table 1.

**Table 1:** Patients’ characteristic for high tension glaucoma (HTG) and normal tension glaucoma (NTG) undergoing combined single-use dual blade goniotomy (Cat-SBG) with range (minimum to maximum)

Parameters	HTG (*n* = 31/38 (82.6%))	NTG (*n* = 7/38 (18.4%))	*P* value
Number of eyes (n)	44 (80%)	11 (20%)	
Sex Female (n) Male (n)	16 (51.6%) 14 (36.8%)	3 (42.9%) 4 (57.1%)	0.733
Right eyes (n) Left eyes (n)	23 (53.3%) 21 (47.7%)	6 (54.5%) 5 (45.5%)	1.0
Mean age ± SD (years)	74 ± 9 (49 to 87)	68 ± 12 (49 to 81)	0.020*
Mean deviation ± SD (dB)	− 8.1 ± 6.8 (− 22.6 to − 0.18)	− 5.9 ± 11.4 (− 23 to − 0.25)	0.569

*Indicates significant difference (*P*  < 0.05)

In all patients, Cat-SBG could be performed as planned after uncomplicated cataract surgery. No patient was considered a failure due to severe complications or needed additional glaucoma surgery during follow-up.

### HTG eyes

**Fig. 2:**
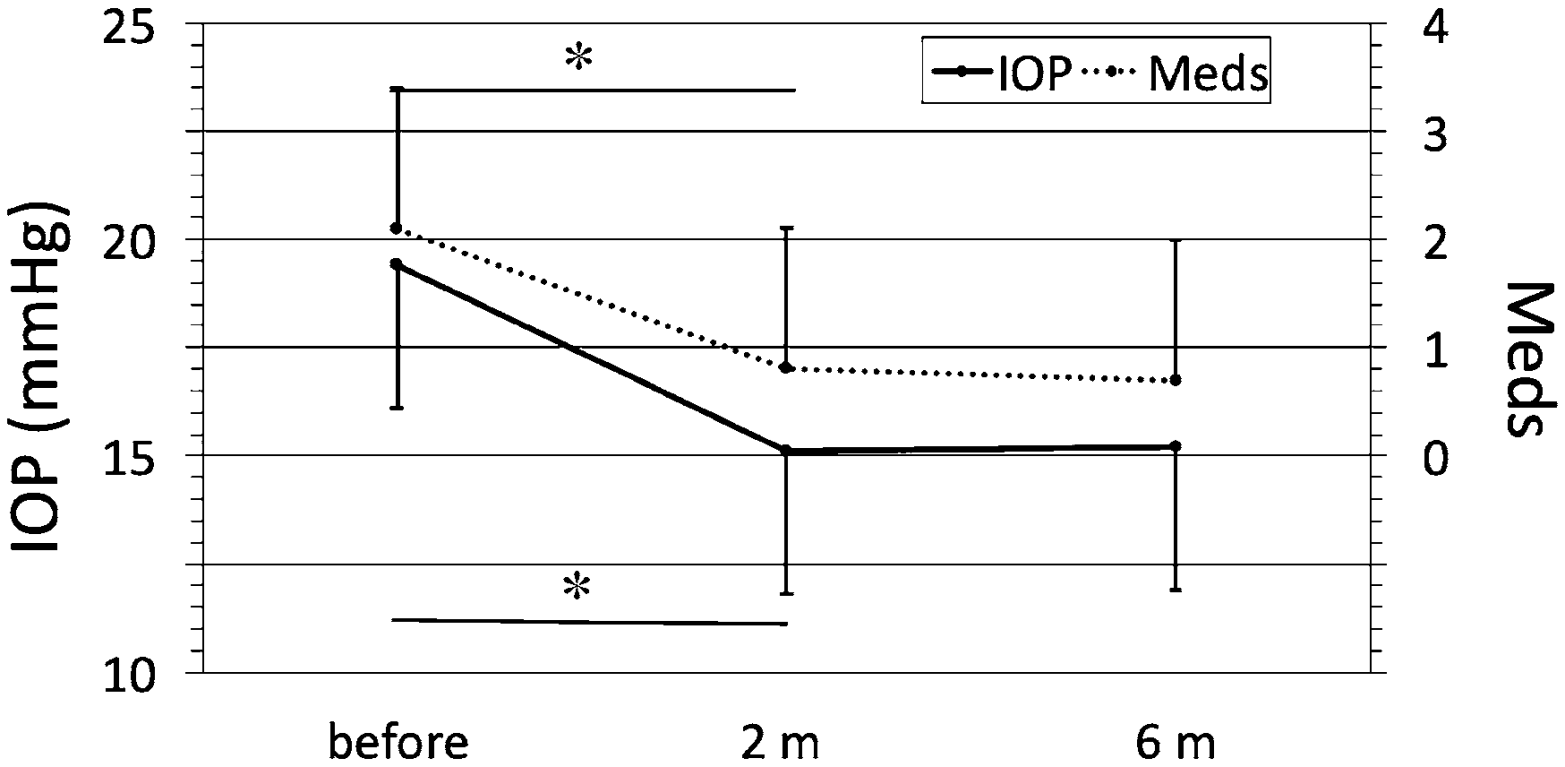
Line graph of the intraocular pressure (IOP, mmHg) and the glaucoma eye drop medication (Meds) in high tension glaucoma (HTG) eyes at different time points following combined cataract surgery with single-use dual blade goniotomy (Cat-SBG): before = before Cat-SBG, 2 m = two months, 6 m = six months after Cat-SBG. Mean IOP and Meds decreased significantly from before to 2 m (*p* < 0.001) with no further significant changes to the 6 m follow-up

Mean IOP before Cat-SBG was 19.4 ± 3.3 mmHg (16 to 29 mmHg) under 2.1 ± 1.3 (0.0 to 4.0) Meds (Fig. 2 and Table 2).

**Table 2:** Intraocular pressure (IOP) and topical glaucoma medication (Meds) before combined cataract surgery and single-use dual blade goniotomy (Cat-SBG) and at different time points after Cat-SBG for high-tension glaucoma (HTG) eyes and normal tension glaucoma (NTG) eyes

	HTG (*n* = 44) before Cat-SBG	HTG (*n* = 44), two months after Cat-SBG	HTG (*n* = 44), six months after Cat-SBG	NTG (*n* = 11) before Cat-SBG	NTG (*n* = 11), two months after Cat-SBG	NTG (*n* = 11), six months after Cat-SBG
IOP ± SD (mmHg)	19.4 ± 3.3	15.1 ± 3.3*	15.2 ± 3.3*	14.0 ± 2.3	11.5 ± 2.3*	12.4 ± 2.3*
Meds ± SD	2.1 ± 1.3	0.8 ± 1.3*	0.7 ± 1.3*	1.6 ± 0.7	0.3 ± 0.7*	0.2 ± 0.7*
Complete success rates (%) for IOP ≤ 21 mmHg (*n* =)	–	55 (24/44)	57 (25/44)	–	(73 (8/11))	(81 (9/11))
Complete success rates (%) for IOP ≤ 18 mmHg (*n* =)	–	50 (22/44)	43 (19/44)	–	73 (8/11)	81 (9/11)
Complete success rates (%) for IOP ≤ 16 mmHg (*n* =)	–	36 (16/44)	36 (16/44)	–	73 (8/11)	81 (9/11)
Complete success rates for ≥ 20% IOP reduction (n =)	–	29 (13/44)	30 (13/44)	–	27 (3/11)	27 (3/11)
Qualified success rates (%) for IOP ≤ 21 mmHg (n =)	–	100(44/44)	100(44/44)	–	(100 (11/11))	(100 (11/11))
Qualified success rates (%) for IOP ≤ 18 mmHg (*n* =)	–	90 (40/44)	93 (41/44)	–	100 (11/11)	100 (11/11)
Qualified success rates for IOP ≤ 16 mmHg (*n* =)	–	65 (29/44)	64 (28/44)	–	100 (11/11)	100 (11/11)
Qualified success rates for ≥ 20% IOP reduction (*n* =)	–	52 (23/44)	43 (19/44)	–	64 (7/11)	27 (3/11)

*Indicates significant changes compared to before Cat-SBG (*p* < 0.05). There were no further significant changes in IOP or Meds between the consecutive follow-up visits

Complete success rates (%) (no topical glaucoma medication (Meds)) and qualified success rates (%) (irrespective of Meds) for IOP levels ≤ 21, ≤ 18, ≤ 16 mmHg as well as ≥ 20% IOP reduction at two and at six months after Cat-SBG for HTG eyes and NTG eyes

*n* = number of eyes

Mean IOP decreased significantly from before to two months after Cat-SBG (IOP 15.1 ± 3.3 mmHg (10 to 20 mmHg); *p* < 0.001). The number of Meds was significantly reduced to 0.8 ± 1.3 (0 to 4.0) (*p* < 0.001) two months after Cat-SBG. IOP and Meds did not increase from two months until six months after Cat-SBG (IOP 15.2 ± 3.3 mmHg (10 to 20 mmHg); *p* = 1.0; Meds 0.7 ± 1.3 (0 to 3.0); *p* = 1.0).

**Fig. 3:**
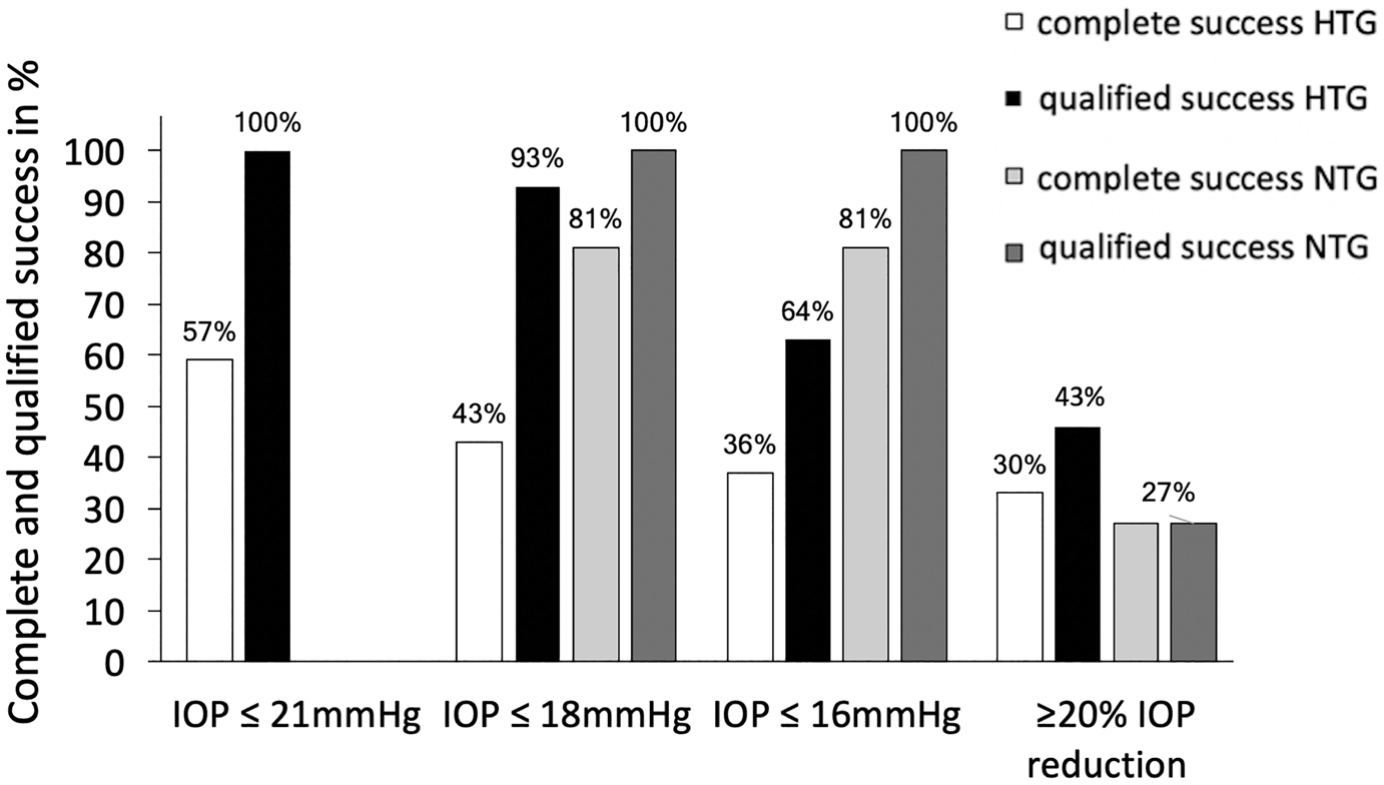
Success rates for high tension glaucoma (HTG) and normal tension glaucoma (NTG) eyes six months after single-use dual blade goniotomy with cataract surgery (Cat-SBG). Success rates for IOP ≤ 21 mmHg in NTG eyes not included

Two months after Cat-SBG, complete success and qualified success were 55% (24/44) and 100% (44/44) for IOP ≤ 21 mmHg, 50% (22/44) and 90% (40/44) for IOP ≤ 18 mmHg, 36% (16/44) and 65% (29/44) for IOP ≤ 16 mmHg as well as 29% (13/44) and 52% (23/44) for ≥ 20% IOP reduction. Six months after Cat-SBG, complete success and qualified success were 57% (25/44) and 100% (44/44) for IOP ≤ 21 mmHg, 43% (19/44) and 93% (41/44) for IOP ≤ 18 mmHg, 36% (16/44) and 64% (28/44) for IOP ≤ 16 mmHg as well as 30% (13/44) and 43% (19/44) for ≥ 20% IOP reduction (Table 2 and Fig. 3).

### NTG eyes

**Fig. 4:**
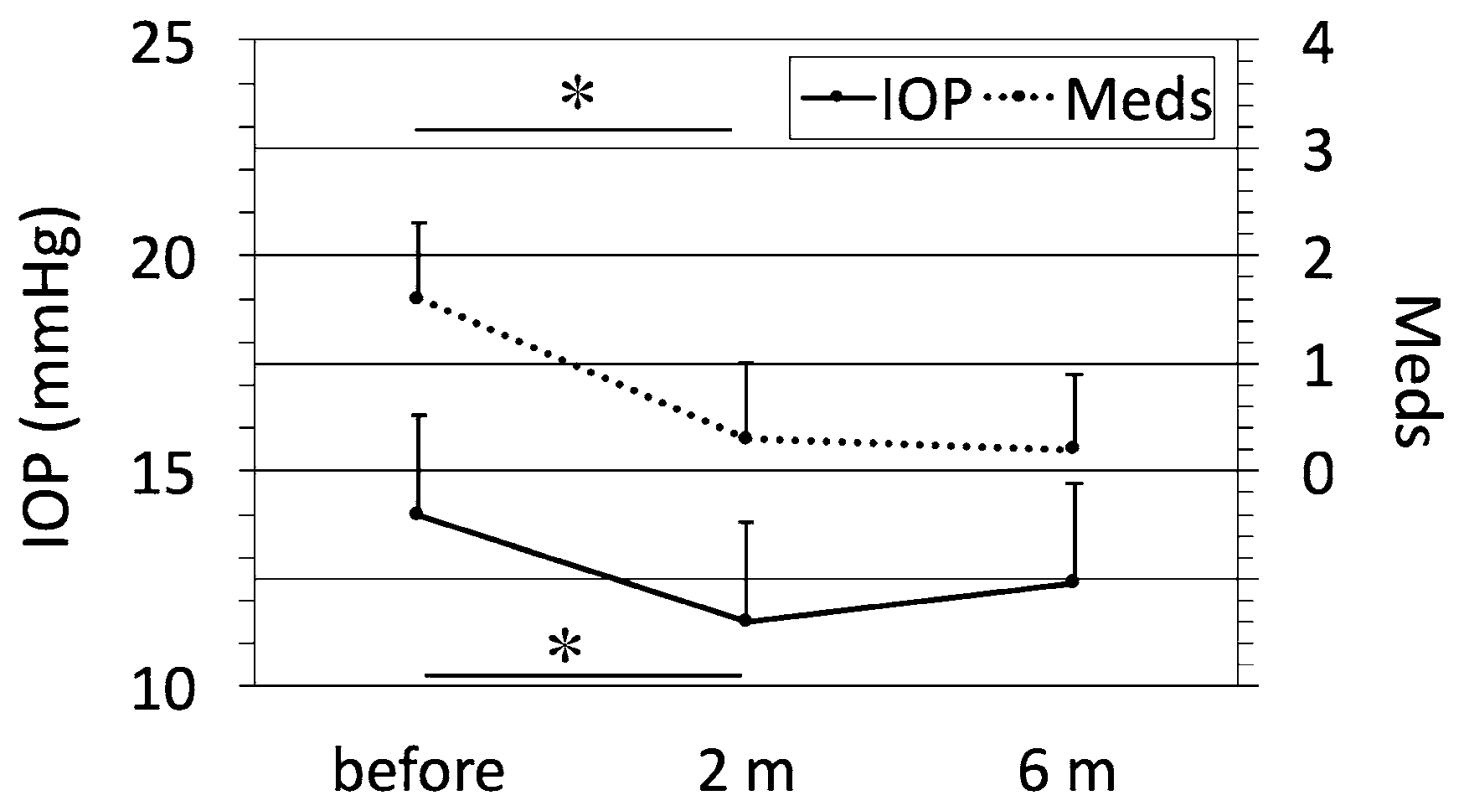
Line graph of the intraocular pressure (IOP, mmHg) and the glaucoma eye drop medication (Meds) in normal tension glaucoma (NTG) eyes at different time points following combined cataract surgery with single-use dual blade goniotomy (Cat-SBG): before = before Cat-SBG, 2 m = two months, 6 m = six months after Cat-SBG. Mean IOP (*p* = 0.004) and Meds (*p* < 0.001) decreased significantly from before to 2 m with no further significant changes to the 6 m follow-up

Mean IOP before Cat-SBG was 14.0 ± 2.3 mmHg (12.0 to 19.0 mmHg) under 1.6 ± 0.7 (1.0 to 4.0) Meds (Table 2 and Fig. 4). Mean IOP decreased significantly from before to two months after Cat-SBG (11.5 ± 2.3 mmHg (9.0 to 15.0 mmHg); *p* = 0.004). The number of Meds was significantly reduced to 0.3 ± 0.7 (0 to 1.0) (*p* < 0.001) two months after Cat-SBG. IOP and Meds did not increase from two until six months after Cat-SBG (IOP 12.4 ± 2.3 mmHg (10.0 to 15.0 mmHg); *p* = 0.706; Meds 0.2 ± 0.7 (0 to 1.0); *p* = 1.0).

Two months after Cat-SBG, complete success and qualified success were 73% (8/11) and 100% (11/11) for IOP (≤ 21), ≤ 18 and ≤ 16 mmHg as well as 27% (3/11) and 64% (7/11) for ≥ 20% IOP reduction. Six months after Cat-SBG, complete success and qualified success were 81% (9/11) and 100% (11/11) for IOP ≤ 18 and ≤ 16 mmHg as well as 27% (3/11) and 27% (3/11) for ≥ 20% IOP reduction (Table 2 and Fig. 3).

### NTG versus HTG

In HTG eyes, the relative IOP reduction was 17.4 ± 18.6% and Meds were reduced by 1.4 ± 1.1 from before to six months after Cat-SBG. The overall IOP reduction was 21% and Meds were reduced by 67%.

In NTG eyes, the relative IOP reduction was 12.6 ± 14.0% and Meds were reduced by 1.4 ± 1.0 from before to six months after Cat-SBG. The overall IOP reduction was 11% and Meds were reduced by 87%.

IOP and Meds reduction showed no significant difference between HTG and NTG eyes at the six-month follow-up (IOP reduction: *p* = 0.425, Meds reduction: *p* = 0.900).

### Complications

No intraoperative complications occurred. Mild anterior chamber bleeding was considered as a sign of successful removal of the trabecular meshwork. Three eyes had a mild to moderate fibrin reaction in the first two weeks after surgery which resolved in all cases under hourly prednisolone acetate 1% eye drop therapy within the next two weeks. In one patient, the fibrinous membrane had to be opened using an yttrium–aluminium-garnet laser (Visulas YAG II, Carl Zeiss Meditec AG, Jena, Germany). Two eyes showed a mild macular edema after surgery: one eye showed the macular edema one week after surgery. Four weeks later, the edema had resolved completely using nepafenac 3 mg/ml eye drops once daily (Nevanac 3 mg/ml, Novartis, Basel, Schweiz) and acetazolamide 250 mg (Glaupax 250 mg, OmniVision GmBH, Puchheim, Germany) twice daily for two weeks. The other eye developed the macular edema four months after surgery, which resolved four weeks later using nepafenac 3 mg/ml eye drops once daily. Three eyes showed a transient IOP spike ≥ 25 mmHg in the first postoperative week. All these eyes had a normotensive IOP at the next follow-up without any further intervention. One eye showed a raised IOP due to a steroid response nine days after Cat-SBG. Prednisolone acetate 1% eye drop therapy was stopped, and loteprednol 0.5% (Lotemax 5 mg/ml, Dr. Gerhard Mann, Berlin, Germany) as well as dorzolamide 2%/timolol 0.5% (Dorzocomp Vision, OmniVision GmbH, Puchheim, Germany) and latanoprost 50 µg/ml (Monoprost, Théa Pharma GmbH Berlin, Germany) were started. At the consecutives follow-ups, IOP was ≤ 21 mmHg.

## Discussion

In this study, we analysed 55 eyes with HTG (*n* = 44) and NTG (*n* = 11) undergoing Cat-SBG. In HTG eyes, we found a significant reduction in IOP (21%) and Meds (67%) from before to six months after surgery leading to complete and qualified success in 43% (19/44) and 93% (41/44) of eyes for an IOP ≤ 18 mmHg.

Separately, we analysed eleven eyes with NTG and found a lesser reduction in IOP (11%) from before to six months after Cat-SBG. Meds were reduced by 87%. When target IOP was defined as ≤ 16 mmHg, complete success and qualified success were 81% (9/11) and 100% (11/11).

No severe intra- or postoperative complications occurred during the six months of follow-up.

In recent years, MIGS procedures have been gaining popularity as they are considered safe, effective and minimally invasive. Ab interno approaches ensure minimal disruption of normal ocular physiology and anatomy as well as a rapid visual recovery [20]. They are routinely performed in mild to moderate glaucoma to reduce Meds and to lower IOP as an early intervention without first exhausting all laser treatments and medication classes [21–24].

Previous studies on Cat-SBG with short (≤ six months) [17, 18, 25, 26] and with longer follow-ups (six to twelve months) showed promising results [12, 14–16, 24, 27]: IOP and Meds were significantly reduced from before surgery to the six- or twelve-month follow-up (see also Table 3).

**Table 3:** Overview of single-use dual blade goniotomy (SBG) publications. Intraocular pressure (IOP), topical glaucoma medication (Meds) before (preop) SBG and six and twelve months postoperatively (postop) with (yes) or without (no) combined cataract surgery (Cat-SBG) or with both variants of operation methods included in the analysis (both)

	*n* =	IOP (mmHg); Meds preop	IOP (mmHg); Meds six months postop	IOP reduction (%) preop to six months postop	Meds reduction preop to six months postop	IOP (mmHg); Meds twelve months postop	Combined cataract surgery
Hirabayashi 2019 [17]	42	17.1 ± 4.8; 2.4 ± 1.3	15.0 ± 3.0; 1.2 ± 1.4	12.3	1.2	–	Yes
Greenwood 2017 [18]	71	17.4 ± 5.2; 1.6 ± 1.3	12.8 ± 2.6; 0.9 ± 1.0 (*n* = 60)	26.4	0.7	–	Yes
Salinas 2018 [26]	52	18.4 ± 6.1; 2.6 ± 1.1	13.9 ± 3.5; 1.4 ± 1.1	24.5	1.2	–	No
Berdahl 2018 [25]	53	23.5 ± 1.1; 2.5 ± 0.2	15.0 ± 0.6; 1.5 ± 0.2	36.2	1	–	No
Dorairaj 2018 [15]	52	16.8 ± 0.6; 1.6 ± 0.2	12.7 ± 0.4; 0.8 ± 0.1	24.4	0.8	12.4 ± 0.3; 0.8 ± 0.1	Yes
Sieck 2018 [12]	197	17.3 ± 0.4; 2.1 ± 0.1	14.2 ± 0.3; 1.4 ± 0.1 (*n* = 162)	17.9	0.7	13.9 ± 0.3;1.6 ± 0.1 (*n* = 140)	Both
Wakil 2020 [16]	116	18.0 ± 6.7; 2.5 ± 1.2	14.5 ± 4.6; 1.5 ± 1.3 (*n* = 85)	19.4	1	14.6 ± 4.8; 1.5 ± 1.3	Both
Kornmann 2019 [14]	111	17.1 ± 4.7; 2.4 ± 1.3	14.1 ± 3.8; 1.0 ± 1.4	17.5	1.4	14.7 ± 3.5; 1.1 ± 1.3 (*n* = 72)	Both
ElMallah 2020 [27]	42	21.6 ± 0.8; 2.5 ± 0.2	14.7 ± 0.7 (*n* = 36); 1.8 ± 0.2	31.9	0.7	16.5 ± 0.8; 2.2 ± 0.2 (*n* = 35)	No

In a recent application study, twelve-month results of 52 patients undergoing Cat-SBG were presented. Here, IOP was reduced from 16.8 ± 0.6 mmHg before the intervention to 12.4 ± 0.3 mmHg (*p* < 0.001) twelve months after surgery which corresponds to an IOP reduction of 26.2% [15]. 63.5% of patients could at least reduce eye drops by one agent [15].

Our study showed comparable results to previous Cat-SBG studies. The lesser IOP reduction in our study compared to the study by El Mallah et al. [27] (31.9%) and Berdahl et al.[25] (36.2%) could be due to the fact that eyes with a higher preoperative IOP are more likely to experience a larger effect on IOP reduction from glaucoma surgery than eyes with lower preoperative IOP [25, 28–30].

New trabeculotomy-derived surgeries [31] include the trabectome procedure [32], the microhook ab interno trabeculotomy [33], the ab interno 360° suture trabeculotomy/gonioscopy-assisted transluminal trabeculotomy [34] and the single-use dual blade goniotomy [11, 31], analysed in this study.

Elevated IOP due to increased resistance to aqueous humour outflow within the TM is considered an important risk factor for POAG [5, 35]. The basic principle of the trabeculotomy techniques is incising or removing TM to lower the outflow resistance, leading to improved IOP levels [31]. The goal of these new techniques is inter alia sparring of conjunctival and scleral incisions, the minimal invasiveness to the ocular surface with less induced postoperative astigmatism and the direct visualization of the anterior-chamber angle with its easier identification of SC compared with identification of TM and SC under the scleral flap during conventional ab externo trabeculotomy [31].

Trabectome is an electrosurgical ab interno procedure that was invented by George Baerveldt and first used in the USA in 2006 [32]. A bipolar 550 kHZ electrode is used to ablate the TM through gonioscopic view [32]. The trabectome system consists of four components: the disposable hand piece, the foot pedal, the cautery generator with a peristaltic irrigation-aspiration and the stand [36]. In a prospective observational study on the outcome of the trabectome (Neomedix, Tustin, CA, USA) in 261 eyes classified as POAG an overall IOP reduction of 25% was reached with a simultaneous reduction in Meds of 43% after a follow-up of 204 ± 278 days [37]. This study showed similar or rather slightly better IOP reduction rates than our study (21%); however, Meds reduction was greater in our study (67%). A pleasant aspect of SBG surgery compared to the trabectome technique is a shorter preparation time as there is no irrigation system that has to be primed and connected [9] and lower costs as the trabectome technology requires the purchase of trabectome equipment that appears to be expensive. Medium-term IOP reduction between the single-use dual blade and trabectome was not differing due to their experience, but long-term results of several years are lacking [9].

Another ab interno technique where a 360° trabeculotomy is performed was recently described by Grover et al. [38]. Hirabayashi and colleagues compared surgical outcomes of 360° circumferential trabeculotomy ab interno (360°Trab, accomplished by Trab360, Sight Sciences, Inc. Menlo Park, CA or gonioscopy-assisted transluminal trabeculotomy (GATT)) versus sectoral excisional goniotomy with the SBG technique at six months [24]. They found out that both SBG and 360°Trab similarly lowered both IOP and the need for IOP-lowering medications during the first six postoperative months [24]. They observed more eyes undergoing SBG than 360°Trab attained target IOP ≤ 18 mmHg (80.0% (56/70) vs 59.3% (16/27), *p* = 0.040) and ≤ 15 mmHg (61.4% (43/70) vs 25.9% (7/27), *p* = 0.003) six months after surgery [24]. They concluded that a full 360° trabecular bypass may not be necessary to achieve maximal efficacy from this class of micro-invasive glaucoma procedures [24].

The majority of trabeculotomy procedures employ a 120-degree trabecular meshwork incision [39]. In our study, 90- to 120-degrees of TM were excised with the majority of procedures having a hundred degrees trabeculotomy.

Another MIGS procedure is the trabecular micro-bypass stent, commonly known as iStent [20]. Aqueous humour drains directly from the anterior chamber into SC bypassing the dysfunctional TM after iStent implantation [40]. Multiple recent studies reported safety and success of the procedure [41–43], and differences in outcome between single versus multiple iStents have been reported [44–46]. A metaanalysis by Popovic et al. reported a significantly higher IOP reduction when implanting two stents compared to one (*p* < 0.001) and a lower postoperative IOP in eyes with two stents compared to eyes with one stent [47].

In comparison with SBG, only a small TM bypass is produced so the question arises how much of TM has to be bypassed or removed and to what extent the transtrabecular flow has to be enhanced to achieve best IOP levels. Further studies have to investigate whether the IOP reducing effect is dependent on the area of “treated” TM and to what extent.

The IOP lowering effect of phaco has been described extensively; thereby, a combined approach of glaucoma surgery and phaco poses an inherent confounding factor [48–50]. Recent studies confirmed the significant IOP reduction after modern phaco cataract surgery and the IOP reduction was generally proportional to the level of preoperative IOP [51–57]. Bhallil et al. found a mean IOP reduction after phaco of 2.3 mmHg in 273 normal patients [58]; Kim et al. reported a mean decrease in IOP of 2.9 mmHg in 31 POAG patients [59].

The additional effect of phaco in Cat-SBG has still to be analysed in further studies.

A particular and important aspect of our study was the separate analysis of Cat-SBG in NTG patients: An IOP reduction of 11% was seen from before to six months after Cat-SGB. This IOP reduction was lesser than in HTG eyes (21%), but did not reach significance. This might be due to the small sample size in our NTG group. The lesser IOP reduction in NTG eyes was expectable though as IOP before Cat-SBG was already low with 14.0 ± 2.3 mmHg in NTG eyes. Meds reduction was 87% which indicates that Cat-SBG is just as well a reasonable procedure in NTG eyes, especially when the aim is to reduce or abolish glaucoma eye drop therapy because of Meds intolerance or patient’s incompliance to use Meds. IOP can be theoretically determined by the Goldman equation, which is IOP = (F/C) + P, where F represents aqueous flow rate, C represents aqueous outflow, and P is the episceral venous pressure [60, 61]. A change or fluctuation in any of these variable is expected to alter the IOP [61]. Presuming a higher outflow resistance in HTG eyes compared to NTG eyes, we expected the SBG effect on IOP reduction being significantly higher in HTG eyes; this trend is to be seen, but our results did not reach significance, most probably due to the small sample size in our NTG group. We think this analysis is important as our study is the first study on Cat-SBG procedures that differentiates and compares success rates in HTG eyes with NTG eyes and that indicates that Cat-SBG might be successful in patients at various levels of preoperative IOP.

The efficacy of MIGS in NTG in general has yet to be established. Most MIGS procedures have not been analysed in NTG eyes. The following two studies are available: a study by Neuhann and Neuhann demonstrated a 21.1% IOP reduction twelve months postoperatively in a subgroup of 18 NTG patients treated with iStent inject with combined phaco (*p* = 0.01) [62]. The proportion of medication-free eyes increased significantly from baseline to twelve months (*p* < 0.001) [62].

The case series by Chang et al. was the first report of a variety of MIGS procedures in 45 NTG patients [30]. IOP decreased from 13.7 to 12.3 mmHg (*p* = 0.041) at 2.5 years, and Meds decreased from 2.0 to 1.1 (*p* < 0.001) at 1.5 years in the overall cohort [30]. Although postoperative IOP was significantly lower at all follow-up visits compared to preoperative IOP the IOP reduction rarely achieved 30% [30]. Furthermore, they analysed a subgroup of 16 patients who underwent two MIGS procedures with different mechanisms of action and found out that Meds reduction at the last follow-up was greater with 68.8% versus 35.7% in patients with single MIGS procedures which strongly trended towards significance (*p* = 0.052) [30]. Further studies on success rates in NTG populations are needed to better understand the role of MIGS in these patients.

Limitations of this study include a small NTG subgroup and only six months of follow-up. Cat-SBG is a new procedure, and long-term results are still lacking. However, we believe reporting early results of a novel surgical approach are important to highlight and further propagate the Cat-SBG method. Future studies with larger Cat-SBG cohorts are planned to perform further subgroup analysis and to identify prognostic factors for this method.

We had two HTG eyes of one patient without any local antiglaucomatous therapy where we performed Cat-SBG surgery. This patient had an absolute intolerance to local glaucoma medication and was referred to us for further treatment without any local Meds. Before surgery the IOP was 21 mmHg in the right and 25 mmHg in the left eye. After combined SBG, the IOP normalized to 14 and 15 mmHg at three months and to 15 and 13 mmHg at six months without Meds. We think it is acceptable to include this patient in our analysis.

Finally, the results of this study are promising and show that Cat-SBG as a minimal-invasive method significantly lowers IOP and Meds up to six-month follow-up in HTG as well as in NTG eyes. No other previous study has specifically analysed Cat-SBG effect in NTG eyes. This study shows that Cat-SBG is efficient in patients at various levels of preoperative IOP.
